# Mapping health, social and health system issues and applying a social accountability inventory to a problem based learning medical curriculum

**DOI:** 10.1080/10872981.2021.2016243

**Published:** 2021-12-27

**Authors:** Dervla Kelly, Sarah Hyde, Mohamed Elhassan Abdalla

**Affiliations:** School of Medicine, Faculty of Education and Health Sciences, and Health Research Institute, University of Limerick, Ireland

**Keywords:** Social accountability, medical education, problem-based learning, curriculum mapping, content analysis

## Abstract

Social accountability is a powerful concept. It is applied to medical education to encourage future doctors to take action to address health inequalities and overlooked health needs of disadvantaged populations. Problem-based learning (PBL) provides an ideal setting to teach medical students about these topics. The objective of this study is to explore how well the components of social accountability are covered in a pre-clinical PBL medical curriculum and to determine the usefulness of an adapted validated social accountability framework. We identified Irish health needs and social issues through a literature review. The retrieved documents were aligned to four values (relevance, equity, cost-effectiveness and quality) from a validated social accountability inventory, to generate a map of social accountability values present in the Irish health system and population. We then used the adapted validated social accountability inventory to evaluate the content of the PBL medical curriculum at an Irish medical school. We identified 45 documents, which upon analysis lead to the identification of health and social issues related to social accountability. 66 pre-clinical PBL cases included demographic, health and psychosocial issues similar to the local population. Analysing along the four social accountability values, the PBL cases demonstrated room for improvement in the equity and relevance domains. Topics for expansion are Traveller health, LGBTI health, alcohol use, climate change and more. Medical educators can use the paper as an example of how to apply this methodology to evaluate PBL cases. Adapting and applying a validated framework is a useful pedagogical exercise to understand established societal values related to social accountability to inform a medical curriculum. We identified opportunities to improve the PBL cases to depict emerging global and social issues.

## Introduction

Since the 1980s, the World Health Organisation (WHO) has fostered the idea of socially accountable medical education and socially accountable medical schools [1]. Social accountability in medical education is described by the WHO as ‘the obligation of medical schools to direct their education, research and service activities towards addressing the priority health concerns of the community, region, and/or nation they have a mandate to serve’ [2]. Embedding social accountability values in medical education is accepted as important as demonstrated in 2010 when there was a ground-breaking agreement called the Global Consensus on Social Accountability of Medical Schools (GCSA) [3]. This agreement involved representatives from 130 medical institutions and organisations. It concludes that context and existing and anticipated health needs of the community should be used to guide medical education strategy, education outcomes and teaching activities.

Since then, there has been substantial expansion and commitment to social accountability in accreditation and awards. For example, the Association of Medical Education in Europe (AMEE) has introduced it as part of the ASPIRE indicators of excellence in medical education [4]. There is a substantial body of literature at an educational strategy level [1,5–9]. Despite the pervasiveness of social accountability rhetoric in medical school strategy documents, the literature fails to detail the day-to-day actions of schools that translate these principles into reality [10]. Deciding what elements of social accountability are included in a curriculum has been identified as an area requiring work [11].

Problem-based learning (PBL) offers a setting to teach social accountability values [3]. A PBL case begins with the presentation of a medical problem in the form of a patient visiting a doctor [12]. The story format of the PBL case prompts students to consider the structure and function of the body, and social and cultural history of the patient [13]. Details in the PBL case such as the patient’s name, ethnicity or patient’s voice, provide social and economic contexts. They can also inadvertently or otherwise, contribute to the hidden curriculum by being contrived, assigning blame to the patient, or presenting a bias in attitude [14,15]. These social and cultural narratives provide an opportunity to explore the messy complexities of human experiences that impact healthcare such as moral, environmental, cultural, political, and economic values.

Updating PBL cases and curriculum mapping are ongoing processes. There are multiple guides and research studies on PBL case design and content [16–19], although all of these typically take a narrow focus on a single social issue. Two previous studies undertook qualitative analysis of PBL cases using a person-centred framework [14,20]. These studies found that the patient perspective is not as ubiquitous as expected [21]. Four previous studies carried out demographic-based needs assessment of the health issues presented in PBL cases [22–25]. These studies highlight evolving health care needs [22–24]. Another study looked at gender stereotypes reinforced in PBL cases from a feminist perspective [26]. All these studies highlight that the needs of society change quickly and keeping curricula up to date is an ongoing task. Furthermore, all these studies take a narrow view of social accountability. No study has taken a value-based, theoretically driven approach to assess PBL content with regard to social accountability.

To convey the complexity of social accountability, in 2020, a specific tool was developed to measure the degree to which social accountability issues are covered in PBL curricula [27]. The inventory is derived from the four common core social values outlined by the WHO in the 1990s: relevance, quality, effectiveness and equity [2]. The inventory is a unique tool as it covers all four components. Its 17 items include demographics of the cases, health and social issues, patient-centeredness, cost-effectiveness, shared decision-making, professionalism and multidisciplinary healthcare teams. This tool has yet to be applied with needs assessment methodologies to evaluate the social accountability content of a PBL curriculum.

This study focuses on how the concepts of social accountability are embedded in PBL in the preclinical years of a medical curriculum, which has received little attention previously. The study seeks to use the validated Social Accountability inventory [27] adapted to the local Irish context by a literature review, which articulated explicit health and social issues within the country of Ireland. The inventory was used to carry out a content analysis of PBL cases used in a medical school curriculum to understand the types of non-biomedical information that are conveyed by the cases. It is believed that the validated conceptual social accountability framework aligned to the local situation will enable educators to identify under-represented topics and illustrate a systematic curriculum-level initiative to integrate social accountability values.

## Methods

### Setting

This study is looks at the School of Medicine-University of Limerick’s PBL curriculum. The first two years of Limerick’s 4-year programme centre on PBL. There are 66 weeks of PBL cases, developed in 2006 and reviewed in 2020.

### Design

The study design is a qualitative content analysis. The primary research question to determine if a locally adapted PBL inventory can be used to assess social accountability values in a curriculum. The study is presented in two stages. The first stage is a document analysis of Irish and Limerick health documents/materials to describe the context where the curriculum is set. The rationale for a thorough literature review was that policy documents and documents from charities and advocacy groups can have some inconsistencies and omissions when standing alone. The review was needed to efficiently organise existing information and provide non-biased data to inform social accountability issues in Ireland. We articulate concise definitions for items in the social accountability framework to create a map of the factors that create health inequalities in Ireland. The second stage is the content analysis of the PBL carried out using the validated social accountability inventory [27], with a secondary objective to compare the change in social accountability-related content from the 2006 cases to the 2020.

### Document search strategy and analysis

The authors hand searched grey literature and Lenus database (an Irish Health repository) to identify key health policy documents/materials in the public domain (government reports, public hearings, intergovernmental reports, non-governmental organisation documents, documents produced by charities) to describe the main health and social issues in the Irish healthcare system. The search strategy was based on the topics of the 17-item social accountability inventory. We also invited public health personnel in relevant organisations to review retrieved documents and provided additional references (see acknowledgements).

A narrative summary and graphic of the key documents is presented. The documents are categorised according to the four domains of the social accountability inventory relevance, quality, cost-effectiveness and quality [27], who derived them from the work of the WHO [2].

### Adaptation of the social accountability inventory

The social accountability inventory provides definition for each of its 17 items. The definitions were reviewed to create operational definitions for our study. The operational definitions were created by DK and reviewed by the co-authors to reach a consensus on their meaning, and resolve any disagreements. Co-author (MEA) lead the development of the 17 items and advised on the fidelity of definitions used in this paper [28]. Specific topics that are priority issues in Ireland were attributed to each item informed by the document analysis (Supplementary Table 1).

**Table 1.** A comparison between social accountability inventory and PBL case content

Inventory item		2020 n (%)	2006 n (%)
1. The case addresses relevant social health concerns	yes	15 (22.7%)	15 (22.7%)
	no	51 (77.3%)	51 (77.3%)
2. The case addresses relevant social determinants	yes	14 (21.2%)	14 (21.2%)
	no	52 (78.8%)	52 (78.8%)
3. The case addresses health promotion and preventative measures	yes	11 (16.7%)	11 (16.7%)
	no	55 (83.3%)	55 (83.3%)
4. The case addresses stakeholders (financially)	yes	2 (3%)	2 (3%)
	no	64 (97%)	64 (97%)
5. The case addresses psychosocial issues	yes	51 (77.3%)	51 (77.3%)
	no	15 (22.7%)	15 (22.7%)
6. The case addresses health system management issues	yes	11 (16.7%)	11 (16.7%)
	no	55 (83.3%)	55 (83.3%)
7. The case addresses medical professionalism	yes	11 (16.7%)	11 (16.7%)
	neither agree nor disagree	55 (83.3%)	55 (83.3%)
8. The case triggers a referral	yes	42 (63.6%)	42 (63.6%)
	no	24 (36.4%)	24 (36.4%)
9. The case addresses the evolving role of doctors	yes	0	0
	no	66 (100%)	66 (100%)
10. The case emphasises the importance of a multidisciplinary approach	yes	21 (31.8%)	21 (31.8%)
	no	45 (68.2%)	45 (68.2%)
11. The case addresses the ethnicity of the patient	yes	4 (6%)	4 (6%)
	no/not applicable	62 (94%)	62 (94%)
12. The problem addresses the socioeconomic aspects of the patient	Yes	3 (4.5%)	3 (4.5%)
	no/not applicable	63 (95.5%)	63 (95.5%)
13. The problem addresses the patient’s age group	Yes	7 (10.6%)	7 (10.6%)
	no/not applicable	59 (89.4%)	59 (89.4%)
14. The problem addresses the patient’s gender	Yes	4 (6%)	4 (6%)
	no/not applicable	62 (94%)	62 (94%)
15. The problem scenario includes under-served, disadvantaged, or vulnerable populations in society	Yes	10 (15.2%)	10 (15.2%)
	no/not applicable	56 (84.8%)	56 (84.8%)
16. The problem scenario includes triggers* for discussing treatment costs and providing alternatives	Agree	2 (3%)	1 (1.5%)
	Disagree	0	0
	Neither agree/disagree	64 (97%)	65 (98.5%)
	Not applicable	0	0
17. The problem scenario includes the concept of ‘person-centred healthcare’	Agree	66 (100%)	66 (100%)
	Disagree	0	0
	Neither agree/ disagree	0	0
	Not applicable	0	0

### Data collection and extraction

All PBL cases used in a recent academic year, 2020/2021 and the first year of the programme at the University, 2006/2007 were retrieved. The school publishes a ‘tutor guide’ containing the relevant material for each case. Data was extracted from each guide for every case using a data extraction spreadsheet. The spreadsheet extracted data for 17 items across four domains; relevance, equity, cost-effectiveness and quality. Free text outlining the details of the content was also recorded for each question. Where the topic of the case had previously been attributed to an item (Supplementary Table 1), this was also recorded.

Each case was reviewed by one author (DK) who decided whether content was present or not. Any uncertainty about classification was discussed with all authors and consensus reached about categorisation of content. During a second round of data extraction, topics were developed from the free-text information to organise the content of the cases. These are presented as priority topics in Ireland in the results section. Further information on the operational definition applied to the Social Accountability Inventory are also described in the results.

### Data analyses

The primary analysis is a comparison between content across all cases and local definitions for each item of the 17 items in the SA framework. Analysis was carried out at domain, item and topic levels. All comparisons are narratively described and presented in a table and graphic. To answer a secondary research question, the case content from 2006 is compared with 2020. The findings are also narratively described. As there is no involvement of human subjects since the cases are fully de-identified educational materials, the study does not need ethical approval.

## Results

### Comparison of case content to social accountability inventory

**Figure 1.**
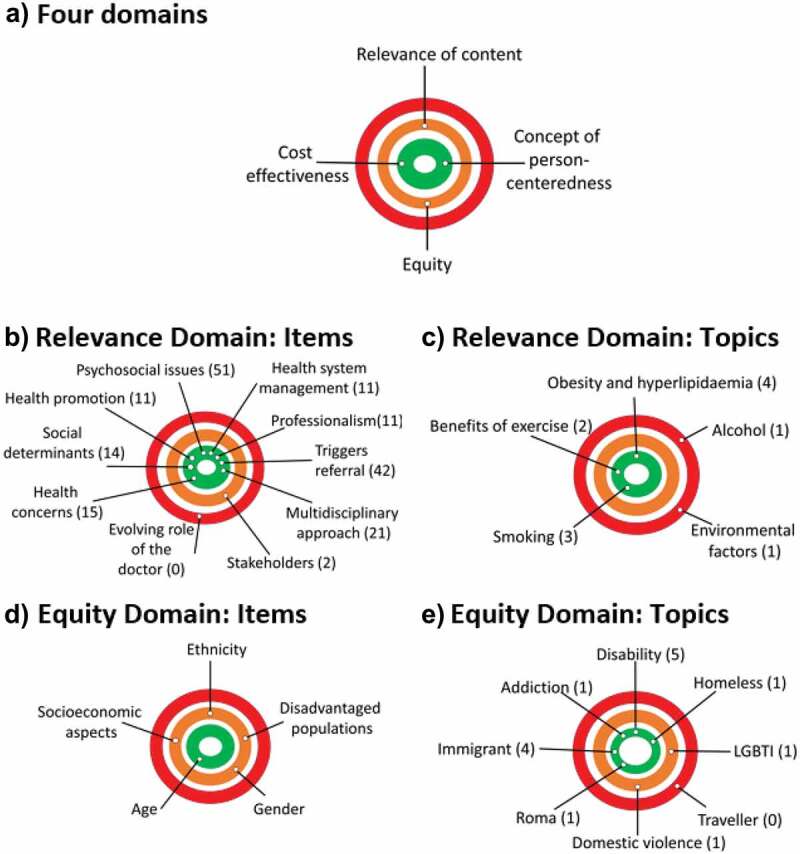
***Areas for improving social accountability content in the PBL cases***. The closer the dot is to the centre of the circle, the better the cases performed in that domain or category. A) Across the 4 domains, there is room for improvement in the equity and relevance of content domains. B) Items from the relevance domain are presented. C) Topics from the relevance domain are presented. There is room for improvement in the area of health education about alcohol and the impact of the environment on health. D) Items from the equity domain are presented. E) Topics from the equity domain are presented. There is room for improvement in the areas of LGBTI and travellers as disadvantaged groups and domestic violence

The domains of quality and cost-effectiveness have content for each indicator and therefore enough content for the domain (Table 1 and Figure 1). In the domains of relevance and equity, there is room for improvement in the case content (Figure 1).

### Relevance

Within the 10 items of the relevance domain, cases adequately address relevant health concerns (22.7%), social determinants of health (21.2%), health promotion and preventative measures (16.7%), psychosocial issues in healthcare (77.4%), health management issues (16.7%), medical professionalism (16.7%), trigger a referral (63.6%) and the importance of a multidisciplinary approach (31.8%). The health promotion content in the cases was frequently presented from a behavioural approach focusing on ‘lifestyle change’. Several cases emphasised that a change in patient’s lifestyle behaviours was central to disease management, with specific references to physical activity, smoking, and managing stress. Over two-thirds of cases identified psychological and social issues related to health ranging from depression to grief to relationships with family and work. For example, several cases discuss the topic of neurophysiology and the focus ranges from biological focuses on managing to pain to psychological and cognitive approaches used to rehabilitation and how approaches vary between health professionals.

Topics identified for further improvement are the area of health education about alcohol and the impact of the environment on health (Figure 1). Regarding alcohol, the consequences of long-term alcohol use are discussed in one case and binge drinking is mentioned in one other case but the focus is on liver function tests, anatomy and physiology of the liver rather than the health promotion and psychological aspects of alcohol use. Very few details related to a patient’s environment such as transport options, access to space for exercise and green spaces were included in the cases.

Item 9, the evolving role of the doctor is not taught in any case (Table 1). Item 4, financial stakeholders in healthcare was included in 3% of cases and highlighted as an area for improvement (Table 1, Figure 1). While the cases regularly identify social supports, the financial aspects of accessing certain management strategies are rarely discussed. One case discusses community services for carers and the role of the GP in accessing caring support. However, issues such as eligibility for a medical card and help from social services and the financial aspects of illness are seldom explored.

### Equity

Within the five items of the equity domain, cases adequately address the ethnicity of the patient (6%) and age (10.6%). Disadvantaged populations (15.2%), gender (6%), and socioeconomic aspects of the patient (4.5%) were considered to have room for improvement with their content. Socioeconomic issues were presented in the cases, although to a lesser extent that age and ethnicity issues. The process of working with interpreters and the difficulties that can arise were explored. Mechanisms by which a patient’s income and education can influence their health were given little attention.

Topics identified for further improvement are the areas of LGBTI and travellers as disadvantaged groups and domestic violence (Figure 1). Regarding gender, the distinction between sex and gender is a learning objective of one case although other issues such as health needs of LGBT across the life course are not discussed at all. Although the issue of child protection is indirectly raised in one case in relation to age of consent and prescribing contraception to a minor, the issue of domestic violence is not discussed. One case raises the issue of bias on the part of healthcare professionals when treating addiction.

### Cost effectiveness

Regarding the third domain of cost-effectiveness, 2% of cases discussed treatment costs and alternatives to treatments with regard to cost (Table 1).

### Quality

For the final domain called quality, which had one item, all cases (100%) were considered to have incorporated values of patient-centeredness (Table 1). Patient-centered care modelled on an integrated care model took the form of reference to a multidisciplinary team and consideration of the emphasis on the patient’s presentation and unique set of circumstances. There was some focus on effective communication strategies, and infrequently cultural issues such as language, religion were mentioned. Overall, the complexities of the non-biological aspects of health management were acknowledged across the cases.

## Change between 2006 and 2020

The main changes between 2006 and 2020 were the localisation of names in 46 cases (69.7%) and changing ethnicities in 5 cases (7.6%) to reflect the Irish population. The topic was changed in one case: a child psychiatry case was changed to include a burn and septicaemia case. The new burn case introduces students to mycology and burn injuries, revisits fluid homeostasis, fluid replacement therapy, renal function, skin function and histology and principles of pain management and reflects on how the death of a child impacts not only on the family but on the healthcare workers involved in the child’s care.

## Documents analysis

45 key documents were analysed and aligned with the four domains of the social accountability inventory (Table 2 and Supplementary Table 2).

**Table 2.** **A summary of the 45 documents retrieved and analysed to map social accountability issues in Irish health services and policy** [Please note some documents apply to more than 1 domain but are entered under the most relevant domain in this table]

RELEVANCE	Burke S, Pentony S. Eliminating health inequalities: A matter of life and death. 2011.
	Limerick Public Participation Network. Limerick Public Participation Network Annual Report 2020. Dublin: Limerick Public Participation Network 2020.
	Organization for Economic Cooperation and Development. Health at a Glance 2019: OECD Indicators. Paris: Organization for Economic Cooperation and Development 2019.
	Farrell C, McAvoy H, Wilde J, Combat Poverty Agency. Tackling Health Inequalities – An All-Ireland Approach to Social Determinants. Dublin: Combat Poverty Agency/Institute of Public Health in Ireland 2008.
	Department of Health. Healthy Ireland Strategic Action Plan 2021–2025: Building on the first seven years of implementation. Dublin: Department of Health 2021.
	Department of Health. Reducing Harm, Supporting Recovery: A Health-led Response to Drug and Alcohol Use in Ireland 2017–2025. Ireland: Department of Health; 2017.
	Department of Health. Healthy Ireland a framework for improved health and wellbeing 2013–2025. Dublin: Department of Health 2013.
	Houses of the Oireachtas. Committee on the future of healthcare: slaintecare report. Dublin: Houses of the oireachtas. 2017.
	Brolcháin NÓ, Britton E, Carlin C, Osagie E, O’Loughlin M, Cormican M et al. Our Environment, Our Health, Our Wellbeing. Ireland: Environmental Protection Agency2021
	Medical Council. Guide to professional conduct and ethics for registered medical practitioners. Dublin: Medical Council 2019.
	Department of Health and Children. 2001. Quality and fairness‐A health system for you, health strategy
	Health Service Executive, Department of Health. eHealth Strategy for Ireland. Dublin: Health Service Executive. 2015.
	Department of Health. Department of Health Statement of Strategy 2021–2023. Dublin: Department of Health. 2021.
	Department of Rural and Community Development. ‘Our Rural Future’ – Rural Development Policy 2021–2025. Dublin: Department of Rural and Community Development 2021.
	Institute of Public Health. IPH response to the Department of Health (ROI) Public Consultation on climate change adaptation for the health sector. Dublin: Institute of Public Health 2019.
	Department of Health. Sharing the Vision: A Mental Health Policy for Everyone. Dublin: Department of Health. 2020.
	Health Service Executive. Performance and Accountability Framework. Dublin: Health Service Executive 2020.
	Department of Health. Health Impacts of Climate Change and the Health Benefits of Climate Change Action: A Review of the Literature. Dublin: Department of Health 2019.
	Government of Ireland. Rebuilding Ireland – Action Plan for Housing and Homelessness. Dublin: Government of Ireland 2016.
	OECD, Union E. Health at a Glance: Europe 2020. 2020.
QUALITY	Department of Health. Sláintecare Implementation Strategy. Dublin: Department of Health. 2018.
	Department of Health. Sláintecare Action Plan 2019. Dublin: Department of Health. 2019.
COST-EFFECTIVENESS	Health Information and Quality Authority. Guidelines for the Budget Impact Analysis of Health Technologies in Ireland. Dublin: HIQA. 2018.
	Health Information and Quality Authority. Guidelines for Evaluating the Clinical Effectiveness of Health Technologies in Ireland. Dublin: HIQA. 2018.
	Health Information and Quality Authority. Guidelines for the Economic Evaluation of Health Technologies in Ireland. Dublin: HIQA; 2020.
EQUITY	Department of Justice and Equality. National Strategy for Women and Girls. Dublin: Department of Justice and Equality 2017.
	Government of Ireland. Roadmap for social inclusion 2020–2025. Dublin: Government of Ireland. 2020.
	Pavee Point. Traveller Health Needs Assessment County Clare: Pavee Point 2019.
	Department of Health. The National Traveller Health Strategy 2002–2005. Dublin: Department of Health. 2002.
	Department of Justice and Equality. National Traveller and Roma Inclusion Strategy 2017–2021. Dublin: Department of Justice and Equality; 2017.
	Pavee Point. Shadow Report to CEDAW Committee: Pavee Point Traveller and Roma Centre 2017.
	Traveller Health Unit. Mid West Community Healthcare Strategic Plan 2018–2022. Limerick: Health Services Executive. 2018.
	Ballyhoura Development CLG. East Limerick Traveller Health Baseline Needs Assessment. Limerick: Ballyhoura Development CLG. 2019.
	All Ireland Traveller Health Study Research Team. 2010. Our Geels: All Ireland Traveller Health Strategy. Dublin. All Ireland Traveller Health Study
	National Women’s Council of Ireland, Department of Health, Health Service Executive. Women’s Mental Health Report. Dublin: National Women’s Council of Ireland 2020.
	Department of Children E, Disability, Integration and Youth. The Migrant Integration Strategy 2017–2020. Dublin: Department of Children, Equality, Disability, Integration and Youth 2019.
	Curran S, Fay R, McGaughey F. Roma in Ireland: a national needs assessment. Dublin: Pavee Point Traveller and Roma Centre 2018.
	McGinnity F, Watson D. Oral Submission to the Joint Committee on Key Issues affecting the Traveller Community. House of the Oireachtas, Dublin. 2021.
	Higgins A, Doyle L, Downes C, Murphy R, Sharek D, DeVries J. The LGBTIreland Report: national study of the mental health and wellbeing of lesbian, gay, bisexual, transgender and intersex people in Ireland. GLEN and BeLonG To; 2016.
	O’Reilly F, Barror S, Hannigan A, Scriver S, Ruane L, MacFarlane A et al. Homelessness: An unhealthy state. Health status, risk behaviours and service utilisation among homeless people in two Irish cities. Partnership for Health Equity. 2015;97.
	Government of Ireland. Comprehensive employment strategy for people with disabilities. Dublin: Government of Ireland 2015.
	AkiDwa, Dorus Luimni, HSE. Migrant Women’s Awareness, Experiences and Perceptions of Health Services in Limerick. Limerick 2012.
	Barrett A, Burke H, Cronin H, Hickey A, Kamiya Y, Kenny RA et al. Fifty plus in Ireland 2011: first results from the Irish Longitudinal Study on Ageing (TILDA). 2011.
	Health Service Executive. LGBT Health: Towards Meeting the Health Care Needs of Lesbian, Gay, Bisexual and Transgender People. Health Service Executive Dublin; 2009.
	Central Statistics Office. 2017. Census 2016 Summary Results, Part 1

### Domain 1: Relevance

Under the domain of relevance, 29 key documents were identified (Figure 1). Key points for each of these topics are outlined below and in Supplementary Table 1.

#### Health issues (Items 1 and 5)

The main causes of mortality in Ireland are cardiology and respiratory related [29]. These include ischaemic heart disease, stroke, pneumonia and COPD. Cancer is also a major cause of mortality, notably lung, colorectal and breast cancer. Diabetes and Alzheimer’s disease are also among the top five causes of mortality and growing in prevalence. For two cancer cases, although the type of cancer does not have a high mortality rate, the material was deemed relevant as it covered staging and treatments relevant to all cancer

Psychosocial issues are a major cause of morbidity [30–32]. Ireland has one of the highest rates (3/36 countries) of mental health illness in Europe with 18.5% of the Irish population recorded as having a mental health illness such as anxiety, bipolar disorder, depression, or alcohol/drug use in 2016 [33].

#### Social determinants of health (Item 2 and 3)

For brevity, socioeconomic, age, gender and disadvantaged groups are discussed in a subsequent section. Environmental factors that impact health in Ireland are air quality, water quality, radon, home and work conditions, neighbourhood condition and access to green spaces [34,35]. Accessing to stable housing is an issue for some groups [36]. The development of integrated care services for those in rural areas is also a notable priority in Ireland [37]. Climate change is another threat to health that increases vulnerability of those already experiences inequalities [38,39].

Risky behaviours such as drinking too much, poor diet and smoking are more common among people with low income or education [29,40]. The Healthy Ireland policy document prioritises reducing inequality as a mechanism to improve overall health [31,41].

#### Health system organisation and issues (Items 4, 6, 8 10)

The Health Service Executive under the Health Service Executive (Governance) Act 2019 manages the performance of the healthcare system, reporting to the government [42]. The Irish health system is financed through general taxation, and all citizens are entitled to public healthcare, however access to this in a timely fashion can be highly variable. The three main stakeholders are the state, individual and insurance companies, 45% of the population choose to pay for additional health insurance [29,33]. Besides the standard healthcare access points [43], private and volunteer-led organisations make a significant dynamic contribution to Limerick’s communities [30,44].

#### Professional roles and responsibilities (Items 7 and 9)

The role of the registered medical professional is set out by the Irish Medical Council’s code of ethics. These principles underpin good care and include trust, collaboration, advocacy, acting as a role model, commitment to lifelong learning and quality improvement to name a few [45].

Healthcare responsibilities evolve because of various local and global forces and the role of doctors will also need to evolve in response. Pertinent issues are demographic changes, organisational factors include the restructuring of Irish healthcare delivery systems, recruiting and retaining doctors, strengthening primary care and addressing hospital capacity are priorities, use of technology, shifting cultural values and new public health prevention and promotion initiatives [29,43,46,47].

### Domain 2: Equity

21 documents related to equity were analysed(Figure 1). Marginalised and underserved groups in Ireland are Travellers, Roma, homeless, people with addiction, people with disability, LGBTI, those with caring responsibilities (often women) and older people [36,48–54].

The degree of disadvantage experienced by Travellers in Ireland is significant and perhaps the greatest of any group [55]. Traveller mortality is 3.5 times higher than non-Travellers are and there are numerous national and local needs assessments and taskforces developing services in this area [56–61].

Gender-specific issues include domestic violence, burden of caring responsibilities and higher rates of self-harm, mental health risks and suicidality among sexual and gender minorities [48,54,62,63].

Undocumented migrants, those seeking asylum and those who have refugee status often experience poor access to health services, training and employment schemes, social protection payments, challenges in accommodation, sanitation and hygiene and lack of culturally appropriate services [51,64]. Language difficulties are an issue for non-English speakers [57,65].

Both bias and hostility towards marginalised groups remains an issue in healthcare services in Ireland [54,57,62]. Regional disparity in service provisions and diversity of needs for minority groups, particularly in rural areas has been noted. Other vulnerable groups in Ireland include the unemployed and those in single-parent households [29,34,66].

### Domain 3: Cost effectiveness

The Department of Health, HSE, the Health Information and Quality Authority and National Centre for Pharmacoeconomics are working on economic measures and policies to increase the availability of newer and cheaper medicines [67–69].

### Domain 4: Quality

Quality centres on the concept of person-centred care. Ireland committed to an integrated care policy concept in 2018 as part of the Sláintecare Programme [70,71] and this was informed by the World Health Organisation framework focused on people-centred actions [72]. Public investment is happening in four Integrated Care Programmes as a priority targeting older people, children, patient flow and prevention and management of chronic disease [71].

## Discussion

### Summary of findings

This is the first paper to measure the extent that social accountability topics are present in a PBL medical curriculum using a validated inventory. The document analysis that informed the inventory was not an exhaustive search of the literature but it did involve an extensive Irish database search (Lenus) and community involvement. As the same issues are repeated across multiple documents, it is unlikely any key issues were omitted. The social accountability inventory domains of quality and cost-effectiveness are adequately considered in the cases, while there is room for improvement in the domains of relevance and equity. Health education about alcohol, the impact of the environment on health, the evolving roles of doctors, financial stakeholders in healthcare, LGBTI, Traveller health, the health implications of domestic violence and socioeconomic influences on health outcomes are areas for improvement in the curriculum. We also found PBL case development over time typically involves small modifications.

### Comparison to the literature

Other techniques for developing socially accountable educational practices have been Boelen and Heck’s (1995) ‘planning, doing, impacting’ framework [2], the CARE model (clinical activity, advocacy, research, education and training) [73], the AIDER model (assess, inquire, deliver, educate, respond) [74], Preston et al.’s (2016) ‘building blocks’ framework, which centres on situational elements encompassing the local health system, workforce and community partners, and the DISCuSS model that emphasises community engagement as a core step in curriculum development [75]. Accordingly, mapping community needs is an established technique to determine a curriculum. Community needs assessments for curriculum development have been previously carried out [76] but this is the first application of the combination of techniques of a PBL specific social accountability inventory together with mapping of local context.

The innovation of the PBL specific inventory is welcome as updating a curriculum requires substantial faculty time and effort. Having a systematic literature review and subsequent checklist of associated topics helps educators ensure PBL cases echo contemporary discourse. A challenge of the literature review is the vast amount of information available nowadays. A critical reflection of topics overlooked and any inconsistencies and biases in the literature is required. Furthermore, the process of identifying local social accountability issues may be bias by faculty opinions, which may not always be resolved by the consensus reaching process applied here. The attitude and time clinician educators and faculty devote to addressing health inequalities can be impeded by narrowly defined learning outcomes, decontextualized assessment methods and already overloaded curricula. Faculty leadership on and commitment to global and social issues is needed for curriculum change to happen [10,77].

The PBL cases in this study are successful in conveying relevant medical issues. Besides the biomedical approach to health, mental health is covered significantly. However, it could be argued that the psychological aspects of the biopsychosocial model such as emotions of shame, guilt, co-dependency behaviours, and clinician/client interpersonal connections could be developed further. A behavioural self-management approach was also strongly represented in the cases. This is similar to previous studies examining PBL case design [14,15,78]. Multidisciplinary input with case development may broaden the conceptualisation and language depicting psychological aspects of health.

PBL case content contained very few triggers to explore environmental issues and wider social issues affecting health. On the one hand, this is not surprising as the primary function of a case is to teach basic science and clinical information [17]. Indeed, the latest World Federation for Medical Education global standards greatly diminish the extent to which health system and population health are required to be covered within a curriculum, designating them optional [79]. The literature also highlights inconsistencies in opinions on the relative importance of global versus local issues when conceptualising social accountability [80,81]. More recently, studies are calling for coverage of global health topics such as climate change in medical curricula [82,83]. The novel coronavirus pandemic has also reinforced the importance of global health. Resources from organisations such as the Global Climate and Health Alliance and the UK Health Alliance on Climate Change could be used [84,85]. It has been demonstrated that ‘bottom up’ student-driven educational initiatives can also work well for emerging health issues [86]. Perhaps encouraging the need for exploration and curiosity about evolving topics can be reinforced during PBL. This would prepare future doctors for the new challenges ahead of them.

Overall, the cases represented the diversity of the Irish population. Promoting social inclusion and the health needs of migrants, LGBTI and Traveller has been operationalised in an Irish health policy context [49,87]. However, it has not been integrated consistently throughout the PBL cases. Overlooking social complexities so that students can focus on the medical condition has been reported previously by MacLeod et al. during her analysis on PBL cases [14]. Involving stakeholders from disadvantaged groups in case design would help articulate their needs and values. This builds on recommendations in the above social accountability models [1,75].

### Implications to research and practice

Reviewing the curriculum using a framework was a useful exercise and supports continuous improvement of the curriculum, a goal set out by the Global Consensus for Social Accountability of Medical Schools [3]. However, there are numerous challenges to creating high-quality PBL cases. Given the heavy workload facing students, identifying curriculum redundancy would be desirable [88]. A study, reviewing social accountability curriculum content across eight medical schools, noted pre-clinical content departs from traditional curricula by having a heavier focus on topics such as health equity, epidemiology and public health ethics, although not necessarily during PBL [9]. Previous work looking at PBL case design considered the complexity and structure of the case, how familiar students were with the topic, whether it interested the students and triggered discussion and critical thinking [89]. Further research should focus on understanding whether new PBL content in areas such as climate change and health in marginalised groups is used as intended in the PBL setting. Important research is also beginning to focus on exploring student perceptions of these issues to understand how to motivate students about social accountability issues [90]. The influence of tutor background on facilitating the social accountability topics in PBL is another area not yet researched in depth [91].

### Strength and weaknesses

This study adapts a robust PBL social accountability inventory following a wide search of grey literature and policy documents to map this information. A limitation of the research is that the PBL cases are curricular teaching materials and do not represent the whole curriculum. That is, something that is missing in a PBL case might be covered in another teaching activity such as a lecture. Additionally, this is a sample of cases from a single institution. A continual audit cycle of teaching across the entire curriculum, covering multiple years and multiple medical schools would bring to light the exact nature of the social accountability curriculum. A limitation of the framework is that it relies on published policy documents and funded research. It may not be representative of issues for people who are not already linked to services or issues that have been well funded to create evidence and policy documents. It is also important to acknowledge the opportunities for exposure to social issues available to medical students through summer projects, electives and more. Finally, this study did not evaluate examination. As assessment drives learning, topics not examined will generally be viewed as non-compulsory by busy medical students, therefore examination standards of the elements social accountability should be worked on as well.

### Conclusion

Social accountability is a broad concept that is often referred to in medical education strategy with less attention paid to how it is operationalised in a medical curriculum. Using a social accountability framework together with a document analysis is useful to determine the degree to which social accountability issues are covered in a PBL curriculum. By applying the framework, we have identified opportunities to improve our curriculum to reflect emerging global and local needs such as Traveller health, LGBTI health needs, alcohol use and the impact of climate change on health. This methodology is a useful tool to maximise the relevance of PBL curriculum to health system and population needs.

## Supplementary Material

Supplemental Material

## References

[1] Preston R, Larkins S, Taylor J, et al. Building blocks for social accountability: a conceptual framework to guide medical schools. BMC Med Educ. 2016;16(1):227. 27565709 10.1186/s12909-016-0741-y PMC5002162

[2] Boelen C, Heck JE, World Health Organization H. Defining and measuring the social accountability of medical schools No. WHO/HRH/95.7 (Geneva: World Health Organisation) . 1995. https://apps.who.int/iris/bitstream/handle/10665/59441/WHO_HRH_95.7.pdf?sequenc.

[3] Global Consensus for Social Accountability of Medical Schools. Global consensus for social accountability of medical schools. Canada: Global Consensus for Social Accountability of Medical Schools; 2010.

[4] ASPIRE. Aspire recognition of excellence in social accountability of a medical school: an Introduction. online: Association for Medical Education in Europe; 2015 https://www.aspire-to-excellence.org/downloads/1303/ASPIRE%20Social%20Accountability%20-%20An%20Introduction_Sept%202017.pdf.

[5] Barber C, van der Vleuten C, Leppink J, et al. Social accountability frameworks and their implications for medical education and program evaluation: a narrative review. Acad Med. Sep 2020. DOI:10.1097/acm.0000000000003731 32910000

[6] Prideaux D. The global–local tension in medical education: turning ‘think global, act local’on its head? Med Educ. 2019;53(1):25–12. 29974492 10.1111/medu.13630

[7] Puschel K, Riquelme A, Sapag J, et al. Academic excellence in Latin America: Social accountability of medical schools. Med Teach. 2020;42(8):929–936. 32503386 10.1080/0142159X.2020.1770712

[8] Bhutta ZA, Chen L, Cohen J, et al. Education of health professionals for the 21st century: a global independent Commission. Lancet. 2010;375(9721):1137–1138. 20362799 10.1016/S0140-6736(10)60450-3

[9] Mullan F, Fair M, Meiri A, et al. Beyond Flexner: a novel framework to implement the social mission of medical education. Educ Health Professions. 2021;4(2):50.

[10] Abdalla ME, Boelen C, Osman WN. Development and evaluation of an online course about the social accountability of medical schools. J Taibah Univ Med Sci. 2019;14(3):241–245. 31435412 10.1016/j.jtumed.2019.03.004 PMC6694871

[11] McCrea ML, Murdoch-Eaton D. How do undergraduate medical students perceive social accountability? Med Teach. 2014;36(10):867–875. 25072172 10.3109/0142159X.2014.916784

[12] Savin Baden M, Wilkie K. Challenging research in problem-based learning. UK: McGraw-Hill Education; 2004.

[13] Schmidt HG. Problem‐based learning: rationale and description. Med Educ. 1983;17(1):11–16. 6823214 10.1111/j.1365-2923.1983.tb01086.x

[14] MacLeod A. Six ways problem-based learning cases can sabotage patient-centered medical education. Acad Med. 2011;86(7):818–25 . 21617504 10.1097/ACM.0b013e31821db670

[15] Hough L, Hegazi I. Challenging the hidden curriculum through problem based learning: a reflection on curricular design. MedEdPublish. 2018;7 3(21): 2312-7996. 10.15694/mep.2018.0000159.1 PMC10701804 38074582

[16] Woodward CA, Ferrier BM. The content of the medical curriculum at McMaster University: graduates’ evaluation of their preparation for postgraduate training. Med Educ. 1983;17(1):54–60. 6823222 10.1111/j.1365-2923.1983.tb01094.x

[17] Azer SA, Peterson R, Guerrero AP, et al. Twelve tips for constructing problem-based learning cases. Med Teach. 2012;34(5):361–367. 22452277 10.3109/0142159X.2011.613500

[18] Sockalingam N, Rotgans J, Schmidt HG. Student and tutor perceptions on attributes of effective problems in problem-based learning. Higher Educ. 2011;62(1):1–16.

[19] Marchais JED. A Delphi technique to identify and evaluate criteria for construction of PBL problems. Med Educ. 1999;33(7):504–508. 10354334 10.1046/j.1365-2923.1999.00377.x

[20] Kenny NP, Beagan BL. The patient as text: a challenge for problem-based learning. Med Educ. 2004;38(10):1071–1079. 15461652 10.1111/j.1365-2929.2004.01956.x

[21] Little P, Everitt H, Williamson I, et al. Observational study of effect of patient centredness and positive approach on outcomes of general practice consultations. Bmj. 2001;323(7318):908–911. 11668137 10.1136/bmj.323.7318.908 PMC58543

[22] Majumder AA. Review of PBL’problems’ and examination questions. Med Teach. 2005;27(5):474. 16231863

[23] Metsemaker JF, Bouhuisj PA, Snellen-Balendong HA. Do we teach what we preach? Comparing the content of a problem-based medical curriculum with primary health care data. Fam Pract. 1991;8(3):195–201. 1959716 10.1093/fampra/8.3.195

[24] Finucane P, Nair B. Is there a problem with the problems in problem-based learning? Med Educ. 2002;36(3):279–281. 11879519 10.1046/j.1365-2923.2002.01150.x

[25] VanLeit B, Cubra J. Student-developed problem-based learning cases: preparing for rural healthcare practice. Educ Health (Abingdon). 2005;18(3):416–426. 16236589 10.1080/13576280500289744

[26] Phillips SP. Problem-based learning in medicine: New curriculum, old stereotypes. Soc Sci Med. 1997;45(3):497–499. 9232743 10.1016/s0277-9536(97)81007-6

[27] Abdalla ME, Dash N, Shorbagi S, et al. Development and validation of inventory tool to evaluate social accountability principles in case scenarios used in problem-based curriculum (Social accountability inventory for PBL). Med Educ Online. 2020;26(1):1847243. 10.1080/10872981.2020.1847243 PMC7737675 33200975

[28] Al Hamid A, Ghaleb M, Aljadhey H, et al. A systematic review of hospitalization resulting from medicine-related problems in adult patients. Br J Clin Pharmacol. 2014;78(2):202–217. 24283967 10.1111/bcp.12293 PMC4137816

[29] Organization for Economic Cooperation and Development. Health at a Glance 2019: OECD Indicators. Paris: Organization for Economic Cooperation and Development; 2019.

[30] Department of Health. Sharing the vision: a mental health policy for everyone. Dublin: Department of Health; 2020.

[31] Department of Health. Healthy Ireland a framework for improved health and wellbeing 2013 – 2025. Dublin: Department of Health; 2013.

[32] Department of Health. Connecting for life. Ireland’s national strategy to reduce suicide 2015–2020. Dublin: Department of Health; 2015.

[33] OECD, Union E. Health at a Glance: Europe 2020. 2020.

[34] Farrell C, McAvoy H, Wilde J. Combat poverty agency. Tackling health inequalities – An all-Ireland approach to social determinants. Dublin: Combat Poverty Agency/Institute of Public Health in Ireland; 2008.

[35] Brolcháin NÓ, Britton E, Carlin C, et al. Our environment, our health, our wellbeing. Ireland: Environmental Protection Agency; 2021.

[36] Government of Ireland. Rebuilding Ireland - action plan for housing and homelessness. Dublin: Government of Ireland; 2016.

[37] Department of Rural and Community Development. ‘Our rural future’ - rural development policy 2021-2025. Dublin: Department of Rural and Community Development; 2021.

[38] Institute of Public Health. IPH response to the department of health (ROI) public consultation on climate change adaptation for the health sector. Dublin: Institute of Public Health; 2019.

[39] Department of Health. Health impacts of climate change and the health benefits of climate change action: a review of the literature. Dublin: Department of Health; 2019.

[40] Department of Health. Reducing harm, supporting recovery: a health-led response to drug and alcohol use in Ireland 2017-2025. Ireland: Department of Health; 2017.

[41] Department of Health. Healthy Ireland strategic action plan 2021–2025: Building on the first seven years of implementation. Dublin: Department of Health; 2021.

[42] Health Service Executive. Performance and accountability framework. Dublin: Health Service Executive; 2020.

[43] Health Service Executive, Department of Health. eHealth strategy for Ireland. Dublin: Health Service Executive; 2015.

[44] Limerick Public Participation Network. Limerick public participation network annual report 2020. Dublin: Limerick Public Participation Network 2020.

[45] Medical Council. Guide to professional conduct and ethics for registered medical practitioners. Dublin: Medical Council; 2019.

[46] Department of Health. Department of health statement of strategy 2021 - 2023. Dublin: Department of Health; 2021.

[47] UN General Assembly. Transforming our world: the 2030 agenda for sustainable development, A/RES/70/12015.

[48] Health Service Executive LGBT Health: Towards Meeting the Health Care Needs of Lesbian, Gay, Bisexual and Transgender People. Dublin: Health Service Executive Dublin; 2009. https://www.drugsandalcohol.ie/12325/.

[49] Government of Ireland. Roadmap for social inclusion 2020-2025. Dublin: Government of Ireland; 2020.

[50] Barrett A, Burke H, Cronin H, et al. Fifty plus in Ireland 2011: first results from the Irish longitudinal study on ageing (TILDA). 2011.

[51] AkiDwa DL. Migrant women’s awareness, experiences and perceptions of health services in limerick. Limerick, Ireland: HSE; 2012. http://doras.org/wp-content/uploads/2014/05/healthmapping.pdf.

[52] Government of Ireland. Comprehensive employment strategy for people with disabilities. Dublin: Government of Ireland; 2015.

[53] O’Reilly F, Barror S, Hannigan A, et al. Homelessness: An unhealthy state. Health status, risk behaviours and service utilisation among homeless people in two Irish cities. (Dublin: Partnership Health Equity). 2015. https://www.drugsandalcohol.ie/24541/.

[54] Higgins A, Doyle L, Downes C, et al. he LGBTIreland Report: national study of the mental health and wellbeing of lesbian, gay, bisexual, transgender and intersex people in Ireland. (Dublin: GLEN and BeLonG To). 2016 https://belongto.org/wp-content/uploads/2018/05/LGBT-Ireland-Full-Reportpdf.pdf.

[55] McGinnity F, Watson D Oral submission to the joint committee on key issues affecting the traveller community. House of the Oireachtas, Dublin. 2021. https://data.oireachtas.ie/ie/oireachtas/committee/dail/33/joint_committee_on_key_issues_affecting_the_traveller_community/submissions/2021/2021-04-20_opening-statement-and-submission-professor-frances-mcginnity-and-professor-dorothy-watson-economic-and-social-research-institute-esri_en.pdf. Cited 2021 Jul 19.

[56] Department of Health. The national traveller health strategy 2002-2005. Dublin: Department of Health; 2002.

[57] Department of Justice and Equality. National traveller and roma inclusion strategy 2017–2021. Dublin: Department of Justice and Equality; 2017.

[58] Pavee Point. Shadow report to CEDAW committee: Pavee Point Traveller and Roma Centre 2017.

[59] Pavee Point. Traveller health needs assessment county. Clare: Pavee Point; 2019.

[60] Traveller Health Unit. Mid west community healthcare strategic plan 2018-2022. Limerick: Health Services Executive; 2018.

[61] Ballyhoura Development CLG. East limerick traveller health baseline needs assessment. Limerick: Ballyhoura Development CLG; 2019.

[62] National Women’s Council of Ireland, Department of health, health service executive. Women’s Mental Health Report. Dublin: National Women’s Council of Ireland 2020.

[63] Department of Justice and Equality. National strategy for women and girls. Dublin: Department of Justice and Equality; 2017.

[64] Department of Children E, Disability, Integration and Youth. The migrant integration strategy 2017-2020. Dublin: Department of Children, Equality, Disability, Integration and Youth; 2019.

[65] Curran S, Fay R, McGaughey F. Roma in Ireland: a national needs assessment. Dublin: Pavee Point Traveller and Roma Centre; 2018.

[66] Burke S, Pentony S Eliminating health inequalities: a matter of life and death 2011.

[67] Health Information and Quality Authority. Guidelines for the budget impact analysis of health technologies in Ireland. Dublin: HIQA; 2018.

[68] Health Information and Quality Authority. Guidelines for evaluating the clinical effectiveness of health technologies in Ireland. Dublin: HIQA; 2018.

[69] Health Information and Quality Authority. Guidelines for the economic evaluation of health technologies in Ireland. Dublin: HIQA; 2020.

[70] Department of Health. Sláintecare implementation strategy. Dublin: Department of Health; 2018.

[71] Department of Health. Sláintecare action plan 2019. Dublin: Department of Health; 2019.

[72] World Health Organization. Integrated care models: an overview. Copenhagan: World Health Organization; 2016.

[73] Meili R, Ganem-Cuenca A, Leung JW-S, et al. The CARE model of social accountability: promoting cultural change. Acad Med. 2011;86(9):1114–1119. 21785308 10.1097/ACM.0b013e318226adf6

[74] Sandhu G, Garcha I, Sleeth J, et al. AIDER: a model for social accountability in medical education and practice. Med Teach. 2013;35(8):e1403–e8. 23444886 10.3109/0142159X.2013.770134

[75] Goez H, Lai H, Rodger J, et al. The DISCuSS model: creating connections between community and curriculum – a new lens for curricular development in support of social accountability. Med Teach. 2020;42(9):1058–1064. 32608298 10.1080/0142159X.2020.1779919

[76] Karimi S, Zohoorparvandeh V. Need assessment of the general practitioner’s curriculum based on clinical activities, advocacy, research, and education (CARE) model. Future Med Educ J. 2019;9(1):18–24.

[77] Ross BM. The socially accountable professor in higher education. J Educ Learn. 2018;7(5):181–187.

[78] Dolmans DHJM, Loyens SMM, Marcq H, et al. Deep and surface learning in problem-based learning: a review of the literature. Adv Health Sci Educ. 2016;21(5):1087–1112. 10.1007/s10459-015-9645-6 PMC5119847 26563722

[79] World Federation for Medical Education. Basic medical education WFME global standards for quality improvement. Copenhagen: WFME; 2020.

[80] Preston R, Larkins S, Taylor J, et al. From personal to global: understandings of social accountability from stakeholders at four medical schools. Med Teach. 2016;38(10):987–994. 26751185 10.3109/0142159X.2015.1114596

[81] Ponka D, Archibald D, Ngan J, et al. Attitudes towards sub-domains of professionalism in medical education: defining social accountability in the globalizing world. Can Med Educ J. 2017;8(2):e37–e47. PMC5669292 29114345

[82] Bandyopadhyay S, Thomas HS, Gurung B, et al. Global health education in medical schools (GHEMS): a national, collaborative study of medical curricula. BMC Med Educ. 2020;20(1):389. 33115465 10.1186/s12909-020-02315-x PMC7594419

[83] Gonzalo JD, Dekhtyar M, Starr SR, et al. Health systems science curricula in undergraduate medical education: identifying and defining a potential curricular framework. Acad Med. 2017;92(1):123–131. 27049541 10.1097/ACM.0000000000001177

[84] The Climate Coalition. The impacts of climate change on public health. UK: The Climate Coalition; 2021.

[85] The Global Climate & Health Alliance . . editor. Health voices on climate change (USA: Adaptation action coalition health launch, Petersberg climate dialogue.). 2021 https://medsocietiesforclimatehealth.org/take-action/health-voices-for-climate-action/.

[86] Hansen M, Rohn S, Moglan E, et al. Promoting climate change issues in medical education: lessons from a student-driven advocacy project in a Canadian medical school. J Climate Change Health. 2021;3:100026.

[87] O’Donnell P, O’Donovan D, Elmusharaf K. Social inclusion in the Irish health context: Policy and stakeholder mapping. J Ir Med Sci. 2020;189(1):11–26. 10.1007/s11845-019-02060-1 31302862

[88] van Merrienboer JJ, Sweller J. Cognitive load theory in health professional education: design principles and strategies. Med Educ. 2010;44(1):85–93. 20078759 10.1111/j.1365-2923.2009.03498.x

[89] Sockalingam N, Rotgans J, Schmidt H. Assessing the quality of problems in problem-based learning. Int J Teach Learn Higher Educ. 2012;24(1):43–51.

[90] Mohammadi M, Bagheri M, Jafari P, et al. Motivating medical students for social accountability in medical schools. J Adv Med Educ Prof. 2020;8(2):90–99. 32426393 10.30476/jamp.2020.84117.1128 PMC7188938

[91] Groves M, Régo P, O’Rourke P. Tutoring in problem-based learning medical curricula: the influence of tutor background and style on effectiveness. BMC Med Educ. 2005;5(1):20. 15938758 10.1186/1472-6920-5-20 PMC1180438

