# Supplementary material for: Mapping health, social and health system issues and applying a social accountability inventory to a problem based learning medical curriculum
Source: Med Educ Online. 2021 Dec 27;27(1):2016243. doi: 10.1080/10872981.2021.2016243 (PMC8725756; doi:10.1080/10872981.2021.2016243)
Supplement: Supplemental Material [file ZMEO_A_2016243_SM7407.docx]

**Supplementary Material:** **Mapping health, social and health system issues and applying a social accountability inventory to a problem based learning medical curriculum**

**Supplementary table 1: Operationalising the social accountability inventory items: an amalgamation of key topics to be covered under each of the 17 items on the PBL social accountability inventory**

| **ITEM** | **Definition** | **Operational definition = topics that are priority issues in Ireland** |
| --- | --- | --- |
| 1. The problem scenario is relevant to social health concerns | *Social health concerns are the major health problems or health issues in the community (country) as identified by appropriate authorities (local health officials, public health bodies, the Ministry of Health, and/or the community)*. | \| Cardiovascular: ischaemic heart disease; stroke; \| \| --- \| \| Respiratory: pneumonia; COPD; \| \| Cancer: lung; colorectal; breast \| \| Diabetes \| \| Alzheimer’s disease \| |
| 2. The problem scenario addresses one or more of the social determinants of health. | *Social determinants of health are economic and social conditions, and their distributions across the population, influencing individual and group differences in health status, such as income, education, employment, etc.* | ** Disadvantaged populations, socioeconomic issues, gender are defined in equity section only for brevity sake  Environmental Factors •Air Quality Index •Water Quality •Radon •Climate change  •Utility affordability  •Transport inequalities |
| 3. The problem scenario points out the relevant principles of health promotion and preventive measures. | *Health promotion integrates the three dimensions of the WHO health definition (physical, social, and mental dimensions, and often spiritual health) that result in* *changed behaviour among individuals and/or the population* | \| - obesity \| \| --- \| \| - alcohol \| \| - smoking \| |
| 4. The problem scenario reflects the involvement of different stakeholders in health care. | *Stakeholders in health include patients, healthcare providers, insurance providers, family, community, society, government, and non-governmental organisations* | 3 main stakeholders:   - State - Individual households - Insurance companies |
| 5. The problem scenario integrates the relevant psychosocial issues, rather than only disease-oriented issues | self-explanatory | Health and social system supports include:  • Support from families, friends and carers  • Access to talk therapies  • Social prescribing  • Digital health interventions  • Community and voluntary group supports  • Peer networks and recovery education  • Home based supports  • Day hospitals  • Crisis house and resolution |
| 6. The problem scenario reflects the relevant health system management issues | *Policies and plans adopted by government and other stakeholders to govern and maintain the health of the communities and individuals; e.g. immunisation policy and infectious disease notifications, etc.*) | Publically funded services of note •National screening service (Breast Check, Cervical Check, Bowel Screen and Diabetic Retina Screen) •Maternal infant and child scheme and immunisations (100% publically funded) •Homecare available on the basis on need (rationed) •37% have free primary care services from GP (means tested) •Publically funded long term residential care: some contribution required and some costs not covered. •Complicated mix of public, private and voluntary care providers: the structural inequality in access to the Irish healthcare system compounds existing inequalities in the health status of the Irish population. |
| 7. The problem scenario includes the relevant elements of medical professionalism | P*rofessionalism is* *the competencies or skills expected of a doctor by the public and individual patients; e.g. honesty and integrity* | Principles:   - trust, collaboration, advocacy, caring, confidentiality, self-care, competence, reflection, acting as a role model, commitment to lifelong learning and quality improvement |
| 8. The problem scenario includes triggers* embedded in the (primary to tertiary) health care referral system based on the case complexity | self-explanatory | Referral systems occur between:   - Primary care - Specialists - Surgery - Pharmacy - Emergency services - Home care - Imaging - Chemotherapy/radiation services - Diagnostic laboratories - Multidisciplinary teams and key workers |
| 9. The problem scenario includes triggers* linked to the evolving roles of doctors in the health system | *The evolving role of doctors in the twenty-first century includes working in teams, utilising resources effectively, providing patient-centred care, advocating for health care systems, and increasing accessibility for patients*)*.* | - Demographic changes: ageing populations, rise of chronic diseases and increased demand and complexity of healthcare services - Organisational factors include the restructuring of Irish healthcare delivery systems outlined in Slaintecare and Healthy Ireland - System capacity: Recruiting and retaining doctors remains an issue. Strengthening primary care and addressing hospital capacity are priorities - Technology: Telemedicine; electronic patient records; delegation and referral and MDT coordination; - Shifting cultural values: Continual flow of information has seen rise in culture of avoiding risk as parallel to a healthy lifestyle; Great potential to evolve issues quickly - Public health prevention and promotion initiatives: Plain packaging for all tobacco products became mandatory for all tobacco products sold as of October 2018; Addressing high obesity rates: Sugars sweetened drinks tax, which was part of the law passed in 2017 that became effective in May 2018; Alcohol Act came into law in October 2018 with the objective of reducing alcohol consumption. The law includes minimum unit pricing on alcohol, restrictions on advertising, warning labels on alcohol products and reduced visibility in mixed trading outlets |
| 10. The problem scenario includes triggers* highlighting the importance of a multidisciplinary approach to patient management | self-explanatory | - Members of MDT - Nurses - Doctors - Psychologist - Occupational Therapist - Speech and Language Therapist - Social Worker - Dietician - Behaviour Therapist - Art Therapist - Music Therapist - Pharmacist |
| 11. The problem addresses the ethnicity of the patient. | self-explanatory | - Majority are white Irish [3,854,226 (82.2%)] - Any Other White background (9.5%) [Polish make up largest of these] - Non-Chinese Asian (1.7%) - Other incl. mixed background (1.5%) - Irish Travellers (30,987) made up 0.7 per cent |
| 12. The problem addresses the socioeconomic aspects of the patient. | self-explanatory | Socio‑Economic Factors •Long‑term unemployment •Jobless Households •Education: Retention & Attainment •Literacy and Numeracy  •Transport inequalities  •Utilities |
| 13. The problem addresses the patient’s age group. | self-explanatory | no adaptation required |
| 14. The problem addresses the patient’s gender. | self-explanatory | no adaptation required |
| 15. The problem scenario includes under-served, disadvantaged, or vulnerable populations in society. | self-explanatory | relevant disadvantaged populations:  travellers, Roma  immigrants, asylum seekers,  people with disability  people with addiction  LGBTI  Homeless |
| 16. The problem scenario includes triggers* for discussing treatment costs and providing alternatives. | self-explanatory | no adaptation required |
| 17. The problem scenario includes the concept of ‘person-centred healthcare’ | *Person-centred care is a way of thinking and doing things that sees the people who use health as equal partners in planning, developing, and monitoring care to make sure it meets their needs* | No adaptions required but further examples provided here to illustrate what was looked for in cases: - exploring the disease and illness experience -understanding the whole person -finding common ground -encorporating prevention and health promotion -patient doctor relationship -therapeutic alliance -patient is a part of the decision making process |

Supplementary Table 2 *Key documents outlining social accountability related issues in Ireland which were used to inform supplementary table 1:* Topics (column 3) were extracted from the documents listed in column 2. Common themes were synthesised across documents to create an amalgamation of frequently described topics. This served as the beginning of adapting the PBL social accountability inventory to the Irish context.

| **Social Accountability Inventory DOMAIN and ITEM** | **Document** | **Significant information** |
| --- | --- | --- |
| Relevance;  Item 1 Social health concerns | OECD/European Observatory on Health Systems and Policies Country Health Profile: Ireland[89] | Identified the main causes of mortality as follows:   \| - Ischaemic heart disease; stroke; \| \| --- \| \| - Respiratory; pneumonia; COPD; \| \| - Cancer: lung; colorectal; breast \| \| - Diabetes \| \| - Alzheimer’s disease \| |
| Relevance;  Item 2 Social determinants of health | Eliminating Health Inequalities – A Matter of Life and Death[64] | From these documents local social determinants were divided into X categories:  1.Socio‑Economic Factors   - Long‑term unemployment - Jobless Households - Education: Retention and attainment - Literacy and Numeracy   2.Environmental Factors   - Air Quality Index - Water Quality - Radon - Neighbourhood condition and access to green spaces - Climate change   *Disadvantaged Groups, Ethnicity, Gender are dealt with in the equity section |
|  | Tackling Health Inequalities[90] |  |
|  | Report of the conference on "Creating connections": health inequalities on the island of Ireland - an agenda for change! |  |
|  | The National Traveller Health Strategy 2002-2005[54] |  |
|  | National Traveller and Roma Inclusion Strategy 2017 – 2021[55] |  |
|  | Climate change and health |  |
|  | IPH response to the Department of Health (ROI) Public Consultation on climate change adaptation for the health sector |  |
|  | Our Environment, Our Health, Our Wellbeing[33] |  |
|  | Rebuilding Ireland - Action Plan for Housing and Homelessness. 2016. Gov of Ireland |  |
|  | 'Our Rural Future' - Rural Development Policy 2021-2025. Depart of Rural and Community Development |  |
|  | Roadmap for social inclusion 2020-2025. Gov of Ireland |  |
| Relevance;  Item 3 Health promotion and prevention | OECD/European Observatory on Health Systems and Policies Country Health Profile: Ireland[89] | Identified the major behavioural issues to be tackled in Ireland as:   - Obesity [18% adults now obese; greater than EU average] - Alcohol [nearly one third of adults reported heavy drinking in 2014, greater than EU average] - Smoking [17% Irish adults smoke] |
|  | Healthy Ireland Framework for Improved Health and Wellbeing 2013- 2025 |  |
|  | Reducing harm, supporting recovery. A health-led response to drug and alcohol use in Ireland 2017 - 2025[38] |  |
|  | Healthy Ireland Strategic Action Plan 2021–2025: Building on the first seven years of implementation |  |
| Relevance,  item 4, stakeholders in healthcare: | ‘Quality and Fairness – A Health System for You’[91] | 3 main stakeholders:   - State - Individual households - Insurance companies   The Irish healthcare system is funded primarily through taxation (73% in 2017), below the EU average for solidarity funding at 79%.  No free point of care access to GPS for the majority of the population, which is unique in western Europe. Individual household spending makes up 12%. Voluntary health insurance accounts for 13% spending, higher than western Europe.  In 2001, medical cards introduced for all over 70-year-olds, which gives them free GP access but there was no focus on universalism.  In 2017, Slaintecare was introduced which was **c**ross-party political consensus on major health reform to deliver a universal, single-tier health service. To date there has been limited improvements to realise the plan. |
|  | Committee on the Future of Healthcare Sláintecare Report[92] |  |
|  | OECD/European Observatory on Health Systems and Policies Country Health Profile: Ireland[89] |  |
| Relevance, item 5, psychosocial issues in healthcare | Sharing the Vision: A Mental Health Policy for Everyone[28] | Ireland has high rates of mental illness in adults: 18.5% of the Irish population recorded as having a mental health illness such as anxiety, bipolar disorder, depression, or alcohol/drug use in 2016.  Most commonly cited tools to improve mental health: exercise, talking to a friend/family, eating healthy food, speak with a counsellor, practice mindfulness/yoga.  Health and social system supports include:   - Support from families, friends and carers - Access to talk therapies - Social prescribing - Digital health interventions - Community and voluntary group supports - Peer networks and recovery education - Home based supports - Day hospitals - Crisis house and resolution |
|  | Connecting for Life – Ireland’s National Strategy to Reduce Suicide 2015-2020[30] |  |
|  | Healthy Ireland Framework for Improved Health and Wellbeing 2013- 2025[29] |  |
|  | Health at a Glance: Europe[31] |  |
|  | Limerick Public Participation Network Annual Report 2020 |  |
| Relevance, item 6, health service management issues | Performance and Accountability Framework[40] | The Health Service Executive under the Health Service Executive (Governance) Act 2019 is accountable to the Minister for Health as well as the government to manage the performance of the healthcare system.  The healthcare system, overseen by the HSE, can be divided into 7 Hospital Groups (including children’s hospital) which represent all the hospitals, nine Community Healthcare Organisations (CHOs) and 6 other national services: the Primary Care Reimbursement Service (PCRS); the National Ambulance Service (NAS), Nursing Home Support Scheme (NHSS), Environmental Health, Public Health and the National Screening Service. |
| Relevance, item 7, professionalism | Guide to Professional Conduct and Ethics for Registered Medical Professionals[43] | Sets out the principles of professional practice that all doctors registered with the Council are expected to follow: trust, collaboration, advocacy, caring, confidentiality, self-care, competence, reflection, acting as a role model, commitment to lifelong learning and quality improvement |
| Relevance, item 8, referral system | eHealth strategy for Ireland [41] | Referral systems occur between:   - Primary care - Specialists - Surgery - Pharmacy - Emergency services - Home care - Imaging - Chemotherapy/radiation services - Diagnostic laboratories - Multidisciplinary teams and key workers   Key priorities in this area are: National Health Identifier Infrastructure, ePrescribing Systems, online Referrals and Scheduling, telehealthcare, development of patient Summary Records and online access to Health Information |
| Relevance, item 9 evolving role of doctors | Transforming our World: The 2030 Agenda for  Sustainable Development[45] | Pertinent changes to healthcare system identified are:   - Demographic changes: ageing populations, rise of chronic diseases and increased demand and complexity of healthcare services - Organisational factors include the restructuring of Irish healthcare delivery systems outlined in Slaintecare and Healthy Ireland - System capacity: Recruiting and retaining doctors remains an issue. Strengthening primary care and addressing hospital capacity are priorities - Technology: Telemedicine; electronic patient records; delegation and referral and MDT coordination; - Shifting cultural values: Continual flow of information has seen rise in culture of avoiding risk as parallel to a healthy lifestyle; Great potential to evolve issues quickly - Public health prevention and promotion initiatives: Plain packaging for all tobacco products became mandatory for all tobacco products sold as of October 2018; Addressing high obesity rates: Sugars sweetened drinks tax, which was part of the law passed in 2017 that became effective in May 2018; Alcohol Act came into law in October 2018 with the objective of reducing alcohol consumption. The law includes minimum unit pricing on alcohol, restrictions on advertising, warning labels on alcohol products and reduced visibility in mixed trading outlets - COVID vaccination and mitigation - Climate change vulnerability: pre-existing health inequalities are at risk of poorer health outcomes related to job losses and environmentally challenging living and working conditions. |
|  | Department of Health Statement of Strategy 2021-2023 |  |
|  | eHealth strategy for Ireland [41] |  |
|  | OECD/European Observatory on Health Systems and Policies Country Health Profile: Ireland[89] |  |
| Relevance, item 10, multidisciplinary team approach | Sláintecare Implementation Strategy[68] | Ireland was late to commit to a comprehensive integrated care policy, only formally adopting one from 2018. MDTs are seen as critical to implementing integrated care.  HSE ICPOP was established in 2016 to improve the health of older people with complex care needs, with the primary objective of supporting them at home. Funding was allocated on a phased basis to 13 pioneer sites located around the country between 2016 and 2018 to establish community-based MDTs and bespoke care pathways.  MDT structures vary across sites and teams. Members may include:   - Nurses - Doctors - Psychologist - Occupational Therapist - Speech and Language Therapist - Social Worker - Dietician - Behaviour Therapist - Art Therapist - Music Therapist - Pharmacist   The activities of the team are brought together using a care plan. This co-ordinates their services and gets the team working together towards a specific set of goals. Sometimes the person has a key worker, who becomes the main point of contact for the person.  An example: 10-Step Integrated Care Framework for Older Persons sets out local governance structures, redefining professional roles and creating innovative ‘bottom-up’ work practices as important in MDT functioning. |
|  | Sláintecare Action Plan 2019[69] |  |
|  | Making a start in Integrated Care for Older Persons – A practical guide to the local implementation of Integrated Care Programmes for Older Persons[93] |  |
|  | A 10 Step Framework to Implement Integrated Care for Older Persons[94] |  |
| Equity, item 11, 12, 13 ,14 Ethnicity, socioeconomic status, age, gender | Census 2016 Summary Results, Part 1[95] | Equity issues related to demographics   - Aging population: expect 25% of population to be older than 65 in 2050 - Domestic violence and burden of caring highlighted as issues predominantly affecting women - LGBT self harm, mental health risks and suicidality highlighted as requiring support - Regional disparity in LGBTI service provisions - Diversity of need within LGBTI community - Lack of cultural competence of healthcare providers - Language difficulties - lack of culturally-appropriate services |
|  | UL Hospitals Implementation Plan 2016-2019[96] |  |
|  | Fifty plus in Ireland 2011: The Irish Longitudinal Study on Ageing (TILDA)[48] |  |
|  | Women’s Mental Health Report |  |
|  | The LGBTIreland Report: National Study of the Mental Health and Wellbeing of Lesbian, Gay, Bisexual, Transgender and Intersex People in Ireland[52] |  |
|  | LGBT Health: Towards meeting the health care needs of lesbian, gay, bisexual and transgender people[46] |  |
|  | Eliminating Health Inequalities – A Matter of Life and Death[64] |  |
|  | Tackling Health Inequalities[90] |  |
|  | National Strategy for Women and Girls 2017 – 2020. Department of Justice and Equality |  |
|  | Roadmap for social inclusion 2020-2025. Gov of Ireland |  |
| Equity, item 15 disadvantaged groups | The National Traveller Health Strategy 2002-2005[54] | Identifies the following groups as among the most  disadvantaged and marginalised people in Ireland:   - Travellers - Roma - Homeless - Addiction - Disability - LGBTI |
|  | National Traveller and Roma Inclusion Strategy 2017 – 2021[55] |  |
|  | Homelessness: An Unhealthy State - Health status, risk behaviours and service utilisation among homeless people in two Irish cities[51] |  |
|  | LGBT Health: Towards meeting the health care needs of lesbian, gay, bisexual and transgender people[46] |  |
|  | Migrant Women’s Awareness, Experiences and Perceptions of Health Services in Limerick[49] |  |
|  | Traveller Health Needs Assessment: County Clare[57] |  |
|  | Our Geels: All Ireland Traveller Health Strategy[97] |  |
|  | Roma Health Needs Assessment. Curran et al. 2018 |  |
|  | The Migrant Integration Srategy 2017-2020. Depart of Children, Equality, Disability, Integration and Youth. |  |
|  | Comprehensive employment strategy for people with disabilities. 2015. Gov of Ireland |  |
|  | East Limerick Traveller Health Baseline Needs Assessment. 2019. Ballyhoura Development CLG |  |
|  | Mid West Community Healthcare Strategic Plan 2018-2022. HSE |  |
| Cost-effectiveness, item 16 | Guidelines for the Economic Evaluation of Health Technologies in Ireland[67] | Health Information and Quality Authority (HIQA) has a statutory remit to evaluate the clinical and cost-effectiveness of health technologies, and provide advice to the Minister for Health and to the Health Service Executive (HSE).  The first set of economic evaluation guidelines i.e. Irish Healthcare Technology Assessment Guidelines were introduced in 2000 and these have been regularly updated.  These guidelines are intended to inform economic evaluations conducted by, or on behalf of the HIQA, the National Centre for Pharmacoeconomics, the Department of Health and HSE, to include health technology suppliers preparing applications for reimbursement. The guidelines are intended to be applicable to all healthcare technologies, including pharmaceuticals, procedures, medical devices, broader public health interventions and service delivery models. |
|  | Guidelines for Evaluating the Clinical Effectiveness of Health Technologies in Ireland[66] |  |
|  | Guidelines for the Budget Impact Analysis of Health Technologies in Ireland[65] |  |
| Quality, item 17 person centred care | Sláintecare Implementation Strategy[68] | Ireland committed to integrated care policy concept in 2018 as part of its ten-year Sláintecare Reform Programme.  There are four Integrated Care Programmes being introduced on a phased basis:   - Integrated Care Programme for Older Persons - Integrated Care Programme for Children - Integrated Care Programme for Patient Flow - Integrated Care Programme for Prevention and Management of Chronic Disease |
|  | Sláintecare Action Plan 2019[69] |  |
